# Genome-Wide Identification of the SQUAMOSA Promoter-Binding Protein-like (SPL) Transcription Factor Family in Sweet Cherry Fruit

**DOI:** 10.3390/ijms24032880

**Published:** 2023-02-02

**Authors:** Yueting Sun, Yanyan Wang, Yuqin Xiao, Xiang Zhang, Bingyang Du, Maihemuti Turupu, Chao Wang, Qisheng Yao, Shilin Gai, Jing Huang, Shi Tong, Tianhong Li

**Affiliations:** Department of Pomology, College of Horticulture, China Agricultural University, Beijing 100193, China

**Keywords:** sweet cherry, transcription factor, SPLs, gene family, plant hormone

## Abstract

Plant-specific SQUAMOSA promoter-binding protein-like (SPL) transcription factors play important regulatory roles during plant growth and development, fruit ripening, inflorescence branching, and biotic and abiotic stresses. However, there have been no identification or systematic studies of the SPL gene family in the sweet cherry. In this study, 12 *SPL* genes were identified in the sweet cherry reference genome, which were distributed over 6 chromosomes and classified into six groups according to phylogenetic relationships with other SPL gene families. Nine *PavSPLs* were highly expressed at green fruit stages and dramatically decreased at the onset of fruit ripening, which implied that they were important regulators during fruit development and ripening. The expression patterns of *PavSPL* genes under ABA, GA, and MeJA treatments showed that the *PavSPLs* were involved in the process of fruit ripening. A subcellular localization experiment proved that PavSPL4 and PavSPL7 proteins were localized in the nucleus. The genome-wide identification of the *SPL* gene family provided new insights while establishing an important foundation for sweet cherry studies.

## 1. Introduction

Transcription factors are an important class of regulatory proteins in gene expression that recognize and bind cis-acting elements to activate or repress the expression of downstream genes. Many plant-specific transcription factors have been identified in plants, such as NAC (NAM, ATAF, and CUC2) WRKY, ARF (auxin response factor), and SPL (SQUAMOSA promoter-binding protein-like), etc. [1].

SPL genes (*AmSBP1* and *AmSBP2*) were first discovered in the floral meristem of *Antirrhinum majus* [2]. Then, cDNA cloning of the *SBP* gene family was performed in *Arabidopsis thaliana* [3]. Subsequently, the *SPL* gene family have been identified in many other plant species, such as 15 SPLs in the tomato (*Solanum lycopersicum* L.) [4], 14 SPLs in woodland the strawberry (*Fragaria vesca*) [5], 27 SPLs in the apple (*Malus domestica* Borkh.) [6], 16 SPLs in the Chinese jujube (*Ziziphus jujuba* Mill.) [7], 16 SPLs in *Arabidopsis,* and 19 SPLs in rice [8]. All SPLs contain a highly conserved DNA-binding domain (also known as SBP domain), which consists of two separate zinc finger structures, which contain Zn-1(Cys3His or Cys4) and Zn-2(Cys2HisCys) [9]. The C-terminus of the SBP domain is rich in basic amino acids, which provide a bidirectional nuclear localization signal (NLS) [10]. As an important transcription factor, the SPL proteins activate or inhibit the expression of target genes by recognizing and binding to a GTAC element in the promoter regions of target genes.

Recently, several functional studies have confirmed the function of SPLs family members among the many plant species. For example, *AtSPL3* was reported to prevent early flowering in *Arabidopsis* [11], *AtSPL9* and *AtSPL15* controlled shoot maturation [12], and *AtSPL9* regulated the vegetative phase change and cell elongation through the BR (brassinosteroid) signaling pathway [13]. *AtSPL8* has recently been identified as affecting GA (gibberellins) signaling [14], while *AtSPL9* negatively regulates anthocyanin accumulation through the destabilization of a MYB-bHLH-WD40 transcriptional activation complex [15]. SBP-box transcription factor tasselsheath-4 (TSH4) was found to regulate bract development and the establishment of meristem boundaries in maize [16]. *SPL* gene-*CNR* (colorless non-ripening) regulates fruit development and ripening in the tomato [17,18,19]. *PySPL9* was found to regulate light-induced anthocyanin biosynthesis in the red pear (*Pyrus pyrifolia* L.) [20]. *VcSPL12* regulates the accumulation of chlorophylls and anthocyanins during fruit ripening in the blueberry (*Vaccinium corymbosum*) [21]. *CpSBP1* modulates fruit softening and carotenoid accumulation by repressing *CpPME1/2* and *CpPDS4* in the papaya (*Carica papaya* L.) [22]. Although SPLs have been shown to be involved in a variety of biological processes, there are limited studies on the function of SPLs in the sweet cherry.

Sweet cherry (*Prunus avium* L.) (2*n* = 2× = 16) is one of the most popular species, having high economic benefits and being widely grown around the world [23]. The sweet cherry shows no respiratory peaks during ripening and little ethylene release, making it a non-respiratory climacteric stone fruit [24,25]. Phytohormones play a vital role in fruit development and ripening. Abscisic acid (ABA) is one of the most important hormones in the ripening process of non-respiratory climacteric fruits [26]. In addition to ABA, other hormones such as auxin, GA, and MeJA (methyl jasmonate) have also been found to be involved in fruit development and ripening [27]. The sweet cherry is known for its excellent taste and various nutrients, such as vitamin C, sugars, organic acids, phenolics, and flavonoids [28]. However, the post-harvest life of the sweet cherry is relatively short, accompanied by darkening of the fruit color and shriveling [29]. Although major progress has been made in pre- and post-harvest treatments to improve the fruit quality and market access, more studies are still needed to obtain high-quality fruit that meets consumer expectations and improves growers’ returns on investment [28].

Although the *SPL* gene family has been identified in many plants, there have been no identification or systematic study reports on the *SPL* gene family in the sweet cherry (*Prunus avium* L.). In this study, we identified 12 characterized putative *SPL* gene family members in the sweet cherry genome. We built a phylogenetic tree of the *SPL* gene family from sweet cherry (*PavSPL*), *Arabidopsis* (*AtSPL*), tomato (*SlySBP* and *CNR*), apple (*MdSBP*), rice (*OsSPL*), and strawberry (*FvSPL*). In addition, we systematically analyzed the gene structure, chromosome location, conserved structural domains, cis-acting elements, and expression patterns of *PavSPL* genes during fruit development and ripening. Furthermore, we characterized the relationships among ABA, GA, and MeJA signaling with *PavSPLs*. These data provide a foundation for the functional analysis of SPL genes in sweet cherry and other species of Rosaceae.

## 2. Results

### 2.1. Identification and Classification of SPL Genes in Sweet Cherry

To identify the *SPL* genes in sweet cherry, all *Arabidopsis thaliana* SPL sequences were used to blast the sweet cherry genome and redundant sequences were removed. The candidate *SPL* genes were verified by the presence of the SBP domain using SMART and Pfam. A total of 12 *SPL* genes named *PavSPL1* to *PavSPL12* according to their chromosomal location by removing genes with incomplete SBP domains (Supplementary Figure S1). The detailed information for the *PavSPL* gene family is given in Table 1, including the protein lengths and accession numbers. The lengths of these 12 SPL proteins varied from 162 (PavSPL7) to 1069 (PavSPL8) amino acids. The predicted MWs ranged from 18 kDa (PavSPL3) to 118 kDa (PavSPL8) (Table 1).

### 2.2. Chromosome Distribution and Synteny Analysis of the PavSPL Family

**Figure 1:**
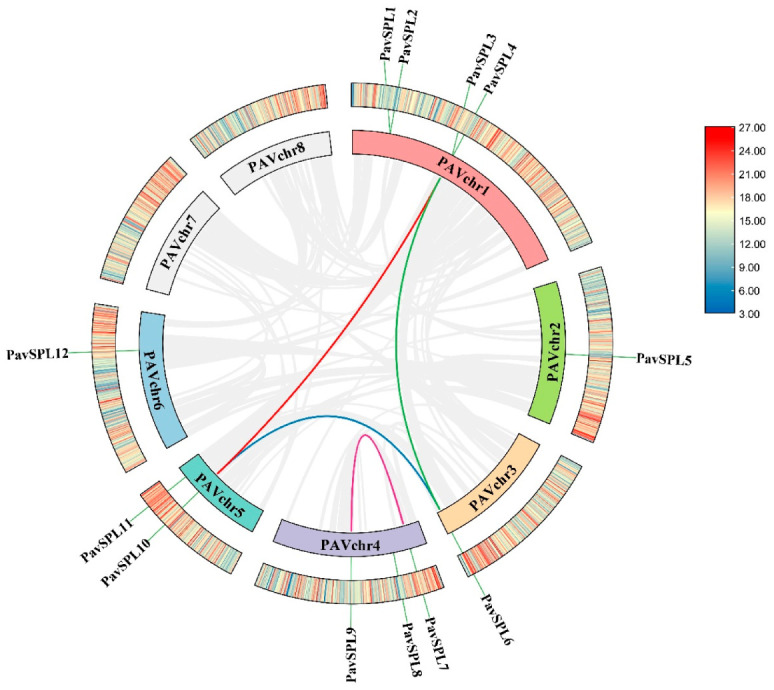
Distribution of the intra-genomic duplications and collinear analysis of *PavSPL* genes in the sweet cherry. Different color lines represent the segmental duplicated *PavSPL* genes. The gray line represents all the covariates, and the chromatic line represents the pairwise replication of the *SPL* gene family.

We constructed a chromosome location and collinearity plot of the sweet cherry *SPL* gene families to further explore the evolutionary relationships among the *SPL* genes. All 12 *PavSPL* genes were located on six of the eight sweet cherry chromosomes, chromosomes 7 and 8 did not contain any *PavSPL* gene, and the most *PavSPL* genes were mapped on chromosome 1 (Figure 1, Supplementary Figure S1). We also used TBtools software v1.106. https://github.com/CJ-Chen/TBtools/releases (accessed on 2 January 2023) to perform an intraspecies covariance analysis and found that the *SPL* gene family was replicated in the sweet cherry genome. The Ka/Ks ratios of all segmental duplicates were less than 1 (Table 2), indicating that the four gene pairs evolved under the effect of purifying selection.

### 2.3. Sequence Alignments and Phylogenetic Analysis of SPL Genes

**Figure 2:**
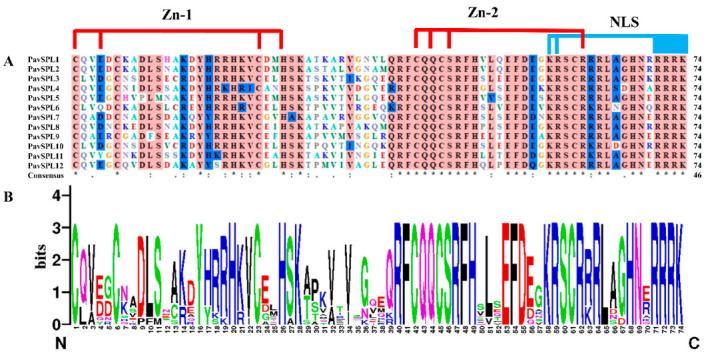
SBP domain alignment of the PavSPL proteins: (**A**) multiple alignments of the SBP domains of the PavSPL proteins; two conserved zinc finger structures (Zn-1, Zn-2) and NLS are shown; (**B**) sequence logos of the SBP domain by the online WebLogo (http://weblogo.berkeley.edu/logo.cgi) (accessed on: 19 November 2022). The overall height of each stack represents the degree of conservation at this position, while the height of the letters within each stack indicates the relative frequency of the corresponding amino acids.

The multiple sequence alignment of full-length proteins was first performed using DNAMAN 9.0 software https://www.lynnon.com/dnaman.html (accessed on: 18 December 2022) to define the protein structure. Multiple alignments of conserved PavSPLs domains in length were completed (Figure 2A). The SBP domains were highly conserved at CQQC, SCR, and RRR sequences with about 74 amino acid residues (Figure 2B). These SPL domains have two conserved zinc finger structures (Zn-1, Zn-2) and a highly conserved nuclear localization signal (NLS).

**Figure 3:**
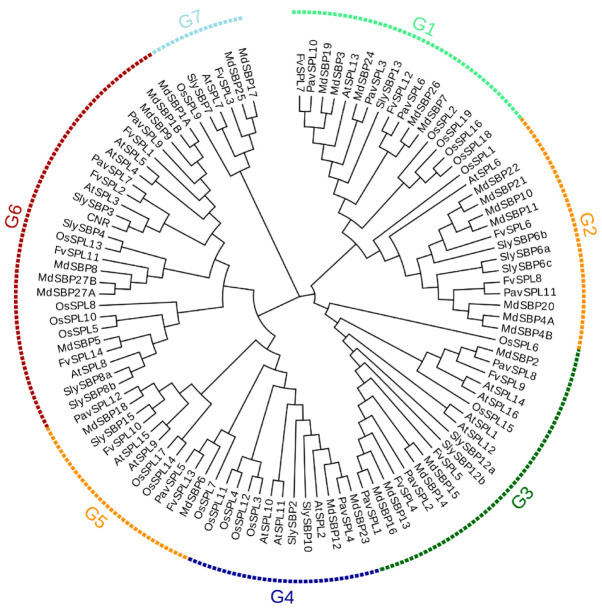
Phylogenetic trees of SPL proteins are from sweet cherry (*PavSPL*), *Arabidopsisa* (*AtSPL*), tomato (*SlySBP* and *CNR*), apple (*MdSBP*), rice (*OsSPL*) and strawberry (*FvSPL*) made by neighbor-joining method with bootstrap values from 1000 replicates. G1, G2, G3, G4, G5, G6 and G7 mean Group1, Group2, Group3, Group4, Group5, Group6 and Group7. The sequence and origin of these plants are shown in Supplementary Table S1.

In order to further understand the evolutionary relationship of PavSPLs, we constructed a phylogenetic tree using the protein sequence of the 12 *PavSPLs*, 16 *AtSPLs*, 15 *SlySBPs* 27 *MdSBPs*, 19 *OsSPLs,* and 14 *FvSPLs* using MEGA 7.0 (Figure 3). All proteins were clustered into seven major groups (named G1 to G7). The 12 PavSPL proteins were clustered into six groups (G1 to G6), and the genes of sweet cherry were not clustered into the seven group. This result was consistent with the classification of *SPL* genes in *Arabidopsis* [3], strawberries [5], and tea (*Camellia sinensis*) [30]. Each group (G1 to G6) contained at least one *PavSPL*. *PavSPL3*, *PavSPL6,* and *PavSPL10* were clustered into G1 together with *AtSPL13*. *PavSPL11* was clustered into G2 together with *AtSPL6*. *PavSPL1*, *PavSPL2,* and *PavSPL8* were grouped into G3 together with *AtSPL1*, *AtSPL12*, *AtSPL14,* and *AtSPL16*. *PavSPL4* was grouped into G4 together with *AtSPL2*, *AtSPL10,* and *AtSPL11*. *PavSPL5* and *PavSPL12* were clustered into G5 together with *AtSPL9* and *AtSPL15*. *PavSPL7* and *PavSPL9* were clustered into G6 together with *AtSPL3*, *AtSPL4*, *AtSPL5,* and *AtSPL8*.

### 2.4. Conserved Motifs and Structure Analysis of the PavSPL Genes

**Figure 4:**
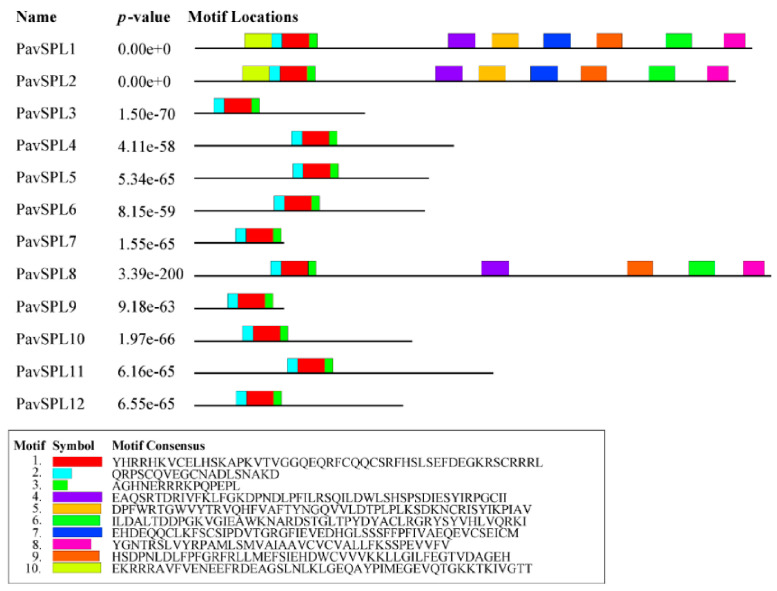
Motif composition of PavSPL proteins. Motifs of PavSPL proteins were analyzed using s MEME analysis. The ten motifs are represented by different colors.

**Figure 5:**
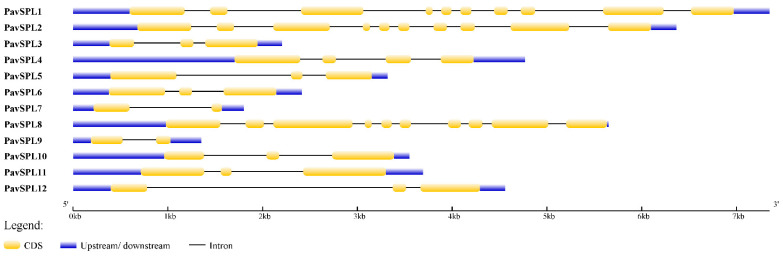
Gene structure of *PavSPL* genes in the online Gene Structure Display Server (GSDS).

The lengths of PavSPL proteins vary widely, ranging from 162 to 1069 amino acids. The MEME motif analysis identified 10 motifs among the PavSPL members in the ‘Zaodaguo’ sweet cherry. Motifs 1, 2, and 3 were annotated as the basic motifs of PavSPL proteins and were present in all PavSPL proteins (Figure 4). This result revealed that motifs 1, 2, and 3 were conserved. Motifs 4, 6, 8, and 9 existed only in PavSPL1, -2, and -8, which were grouped into G3, and the coding sequence (CDS) lengths of these subfamilies were all over 3000 bp. The gene structure prediction showed that all *PavSPL* genes contained exons and introns (Figure 5). The numbers of exons and introns were not conserved among the genes and ranged from one to nine. For example, *PavSPL7* had one intron, whereas the genes in group 3 had nine exons. *PavSPL* genes containing 2 introns were the most common type, accounting for 41.7%.

### 2.5. Cis-Acting Elements in the Promoters of PavSPL Genes

**Figure 6:**
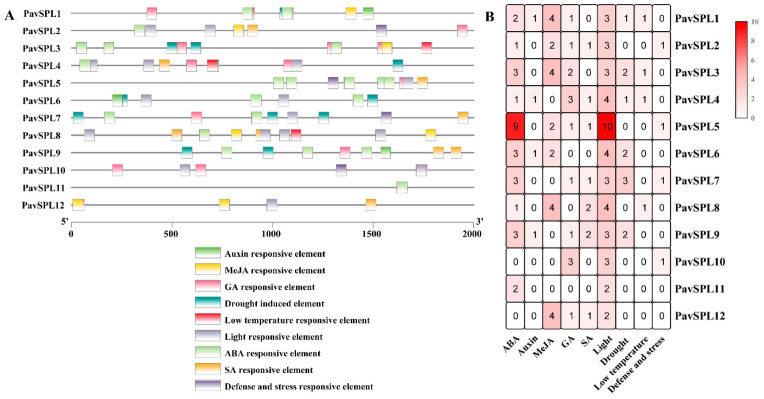
Cis-element of the *PavSPL* genes in sweet cherry: (**A**) the cis-element analysis of the *PavSPL* genes, where colorful boxes represent different cis-elements; (**B**) numbers of cis-elements in the *PavSPL* genes.

Many cis-acting elements on the promoter were distributed upstream of the gene coding sequence, which can regulate the expression characteristics of the genes. Predicting hormone response elements, stress response elements, and light response elements on the are 2000 bp upstream of the *PavSPL* promoter sequence (Figure 6). All 12 *PavSPLs* promoter sequences have hormone response elements, which may play a critical role in the hormone balance in sweet cherries. The element corresponded to five phytohormones, including ABA, MeJA, salicylic acid (SA), GA, and auxin. Most were ABA response elements, indicating that the *PavSPL* genes played a role in the ABA responses.

### 2.6. Expression Analysis of the PavSPL Genes in Different Tissues and in Fruit Development

**Figure 7:**
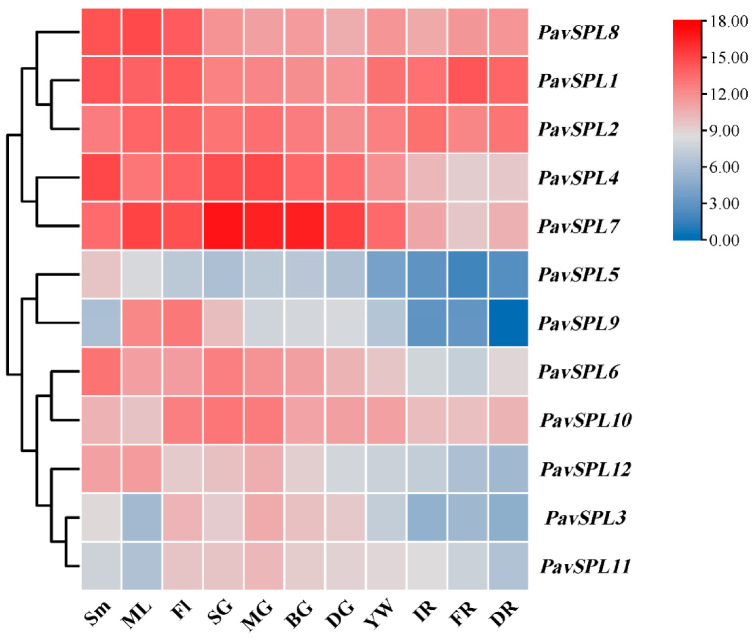
The expression quantities of *PavSPLs* in different tissues as assessed using TBtools. Sm: stems; ML: mature leaves; SG: small green; MG: mid green; BG: big green fruit; DG: de-greening; YW: yellow fruit; IR: initial red; FR: full red fruit; DR: dark red. The relative mRNA levels are represented as the means ± SDs (*n* = 3). Statistically significant differences were assessed using Student’s *t*-test.

To further confirm the function of *PavSPLs* during vegetative and reproductive growth in sweet cherry, we detected the expression patterns of 12 *PavSPL* genes via qRT-PCR testing (Figure 7). The results indicated that 12 *PavSPL* genes displayed diverse expression patterns among the tissues and organs of the sweet cherries. The expression profiles of *PavSPLs* can be divided into two types. Parts of these genes exhibited highly tissue-specific expression patterns, while other parts were structurally expressed in all tissues and organs. For example, *PavSPL1*, *PavSPL2*, *PavSPL4*, *PavSPL7,* and *PavSPL8* exhibited relatively high expression levels in all tissues. *PavSPL9* was specifically expressed in mature leaves and flowers. *PavSPL3*, *PavSPL10,* and *PavSPL11* exhibited higher relative expression levels in reproductive tissues than stems or mature leaves. *PavSPL5*, *PavSPL8,* and *PavSPL12* exhibited higher relative expression levels in vegetative tissues than reproductive tissues.

With the development and ripening of the sweet cherry fruit, the expression levels of *PavSPL3*, *PavSPL4*, *PavSPL5*, *PavSPL6*, *PavSPL7*, *PavSPL9*, *PavSPL10*, *PavSPL11,* and *PavSPL12* decreased gradually; the levels were the highest in the green stage. The expression levels of *PavSPL1* were the highest in the full red stage, and the expression levels of *PavSPL2* and *PavSPL8* remained stable throughout the development stages.

### 2.7. Expression Analysis of the PavSPL Genes during Hormone Treatment

**Figure 8:**
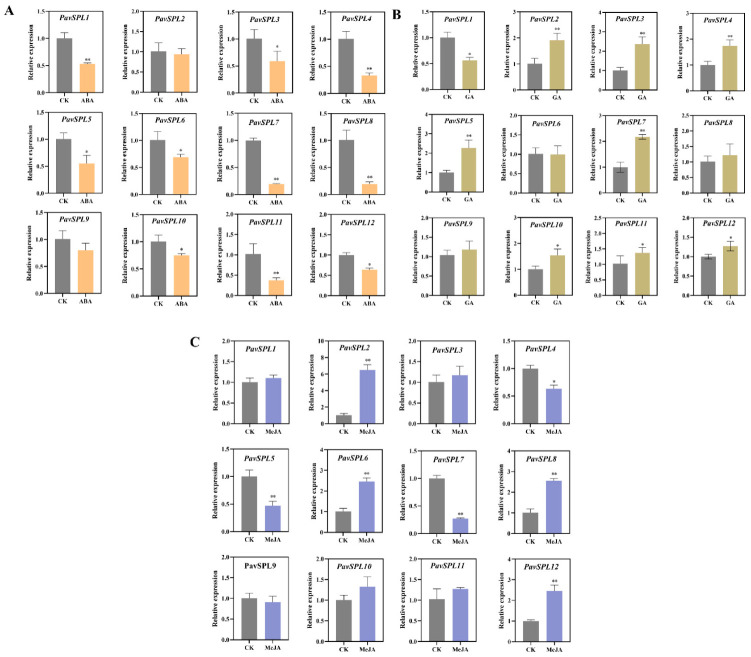
(**A**) ABA treatment, (**B**) GA treatment, (**C**) MeJA treatment. Expression patterns of *PavSPL* genes under ABA, GA, and MeJA treatments. The relative mRNA levels are represented as the means ± SDs (*n* = 3). Statistically significant differences were assessed using Student’s *t*-test (* *p* < 0.05, ** *p* < 0.01).

ABA, MeJA, and GA are the main hormones involved in fruit development and ripening. Therefore, we treated sweet cherry fruits with these hormones and examined the expression levels of *PavSPL* genes. The expression level of *PavSPL1*, *PavSPL3*, *PavSPL4*, *PavSPL5*, *PavSPL6*, *PavSPL7*, *PavSPL8*, *PavSPL10*, *PavSPL11,* and *PavSPL12* were significantly downregulated under the ABA treatment (Figure 8A). Under the GA treatment, *PavSPL2*, *PavSPL3, PavSPL4, PavSPL5*, *PavSPL7*, *PavSPL10*, *PavSPL11*, and *PavSPL12* were significantly induced, while only *PavSPL1* was significantly upregulated (Figure 8B). Under the MeJA treatment, *PavSPL4*, *PavSPL5,* and *PavSPL7* were significantly reduced, while *PavSPL2*, *PavSPL6*, *PavSPL8,* and *PavSPL12* were significantly upregulated (Figure 8C).

### 2.8. Subcellular Localization of PavSPLs

**Figure 9:**
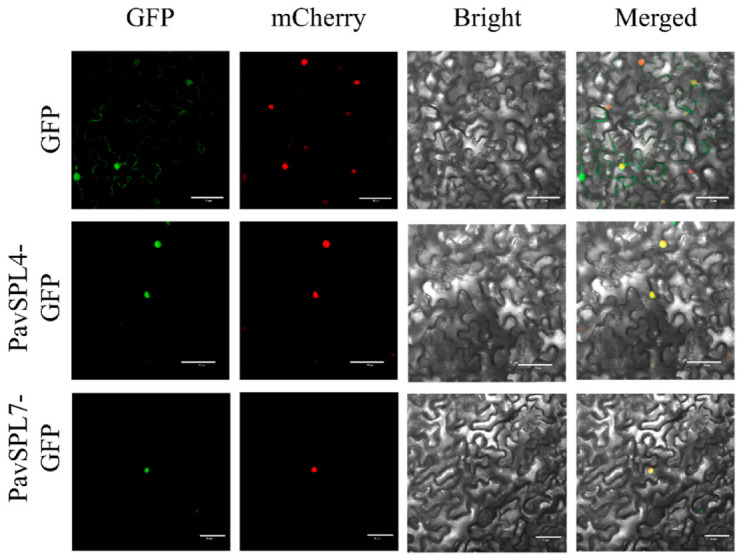
Subcellular localization of PavSPL4 and PavSPL7 proteins. The PavSPL–GFP fusion proteins were transiently expressed in *N. benthamiana* leaves and the GFP fluorescence signal was observed after 48 h. Scale bar, 50 µm.

The nuclear localization of transcription factors is a very important signature. In other species such as VpSBP11 in grape [31] and CpSBP1 in papaya [22], they are localized in the nucleus. In order to investigate the location of *PavSPL* genes in sweet cherry, we examined the subcellular localization of PavSPL4 and PavSPL7 through transient expression in tobacco (*N. benthamiana*) leaves. We found that the green fluorescent protein (GFP) signals of PavSPL4 and PavSPL7 overlapped with the nuclear marker mCherry signal, which indicated that the PavSPL4 and PavSPL7 proteins were localized in the nucleus (Figure 9). These results demonstrated that PavSPL4 and PavSPL7 are nuclear proteins that could serve as transcription factors.

## 3. Discussion

### 3.1. Bioinformatics Analysis of SPL Gene Family in Sweet Cherry

SPL transcription factors have been attracting attention in the scientific community due to their powerful functions. In recent years, although the function of *SPL* genes has been increasingly studied in many plants, it has not been studied in the sweet cherry. In this study, we performed a comprehensive analysis of the *SPL* gene family in the sweet cherry. Firstly, we identified 18 *PavSPL* sequences from the *Prunus avium* genome, which was released in 2017. Among them, *Pav_sc0001411.1_g180.1.mk*, *Pav_sc0001280.1_g590.1.mk*, *Pav_sc0000492.1_g530.1.mk*, *Pav_co4005735.1_g010.1.mk*, *Pav_sc0000815.1_g180.1.mk,* and *Pav_sc0001974.1_g370.1.mk* were excluded for their incomplete SBP domains. The numbers of SPL family members vary greatly among species. For example, there are 16, 25, 24, 29, and 59 members in *Arabidopsis thaliana* [8], tea [30], tartary buckwheat (*Fagopyrum tataricum*) [32], Chinese cabbage (*Brassica rapa* subsp. *pekinensis*) [33], and cotton (*Gossypium hirsutum*) [34], respectively. In this study, 12 *SPL* gene family members were identified from the sweet cherry genome, which was less than for other species, suggesting that *SPL* genes have undergone extensive changes during their genome evolution, which has led to the diversification of the gene functions.

Based on a phylogenetic analysis (Figure 3), the *PavSPL* genes were clustered into six groups, with at least one gene from *Arabidopsis* and the tomato in each group, implying the functional conservation of *SPL* genes between different plants. Members of each subfamily contained a typical SBP domain, which suggested that the SBP domains were conserved. The analysis of the gene structure of the *PavSPL* gene family showed that the maximum numbers of exons and introns were present in G3 (Figure 4 and Figure 5). The minimum numbers of exons and introns were present in G6. These results indicated that the evolution of the *SPL* gene family may be closely related to the diversification of the gene structures [7]. Therefore, we speculate that different SPL groups probably perform similar functions within *Arabidopsis*, due to the presence of similar structures and conserved motifs.

### 3.2. Expression Profiles of PavSPLs in Sweet Cherry Development

*SPL* genes have been reported to be involved in reproductive growth and vegetative growth, but no studies have been performed in sweet cherry. We predicted the function of the *PavSPL* genes based on expression patterns among the tissues and organs of sweet cherry and the functions of homologous genes (Figure 7). G1 contained *PavSPL3*, *PavSPL6*, *PavSPL10,* and *AtSPL13.* These genes had relatively high transcript levels in the stems and flowers, suggesting that they may play vital roles in shoot and flower development. In G3, *AtSPL16*, *PavSPL1*, *PavSPL2,* and *PavSPL8* are similar to *AtSPL1*, *AtSPL12*, and *AtSPL14* and are long genes. Similar to Arabidopsis, these three *PavSPLs* were highly constitutively expressed in all tissues and organs [3]. *AtSPL9* and *AtSPL15* clustered in G5 and have been proved to control shoot maturation redundantly [12]. *PavSPL5* and *PavSPL12* were clustered in G5 and may play the same roles as their presumed orthologs.

Sweet cherry is highly nutritious and the fruit is rich in anthocyanins, with antioxidant activity [35]. The growth and development of sweet cherry is regulated by a variety of transcription factors [35,36,37]. Therefore, studying the expression patterns of *PavSPL* genes in sweet cherries at different stages of development and ripening can provide a basis for screening potential transcription factors that regulate fruit growth and development. The qRT-PCR assay revealed that 12 *PavSPL* genes were expressed in different patterns in the processes of fruit development and ripening (Figure 7), suggesting that *PavSPL* genes may be critical during fruit growth, development, and ripening.

The *SPL* genes play key roles in the fruit development and ripening processes, which have been demonstrated in other species. Different members of the SPL family show functional differences. For example, *VcSPL12* (classified into the same subfamily as *AtSPL2*, *AtSPL10,* and *AtSPL11*) regulated the accumulation of chlorophylls and anthocyanins during fruit ripening in blueberries [21]. Accordingly, *PavSPL4* clustered in G4 with *AtSPL2*, *AtSPL10,* and *AtSPL11* and may have important functions in leaf development or fruit ripening. In papaya, *CpSBP1* (classified into the same subfamily with *SlSPL-CNR, AtSPL3,* and *AtSPL4*) modulated fruit softening and carotenoid accumulation [22]. *SlSPL-CNR* is a key gene that controls tomato fruit ripening. The epigenetic variation in the promoter region of *SlSPL-CNR* affects the ripening of fruit [4,19,38]. *SlSPL-CNR* clustered in G6 with *PavSPL7* and *PavSPL9*. Therefore, *PavSPL7* and *PavSPL9* may have an important function in fruit ripening. They had the same motif compositions, but the specific functions of these *PavSPL* genes need to be verified in further experiments in the future. In our study, we demonstrated that most *SPL* genes such as *PavSPL4* and *PavSPL7* showed a downregulation trend, consistent with the results presented in grapes [39]. This means that these genes are negatively regulated during fruit development and ripening in sweet cherry.

### 3.3. Expression Profiles of PavSPLs in Plant Responses to Multiple Hormones

The hormone signals and environmental changes are critical to fruit development and ripening [40,41,42,43]. In our study, the analysis of cis-acting elements in the 2000 bp sequence upstream of the *PavSPLs* promoter revealed that most members of this family respond to a variety of hormones and stresses, indicating that *PavSPLs* may be regulated by light, stresses, and phytohormones (Figure 6). The roles of SPL genes in hormone regulation are not well defined, but there are some relevant reports in other species. For example, *SPL8* acted as a local regulator in a subset of GA-mediated developmental processes in *Arabidopsis* [14]. SPL9 interacted with ABI5 to enhance the ABA responses in *Arabidopsis* [44]. ELONGATED HYPOCOTYL5 (HY5) interacted with SPL7 to mediate coordinated responses to light and copper [45]. BRASSINAZOLE-RESISTANT 1 (BZR1), the master transcription factor of the BR signaling pathway, interacted with AtSPL9 to cooperatively regulate the vegetative phase change and cell elongation [13]. In previous studies, an exogenous ABA treatment promoted the fruit ripening process and the ABA content increased significantly during fruit ripening in ‘Hong Deng’ and ‘Rainier’ sweet cherries [35,46]. The exogenous ABA treatment could regulate strawberry fruit ripening [47]. In our study, we treated ‘Zaodaguo’ sweet cherries using ABA, GA, and MeJA exogenous hormones. The gene expression pattern showed that the *SPL* genes were responsive to many hormone treatments (Figure 8). *PavSPL4*, *PavSPL5*, *PavSPL7,* and *PavSPL12* were regulated by many hormone treatments, suggesting that the *PavSPL* genes may be involved in the crosstalk of multiple different hormone signals regulating fruit development and ripening.

## 4. Materials and Methods

### 4.1. Plant Materials and Hormone Treatments

The sweet cherry (*Prunus avium* L.) trees of the cultivar ‘Zaodaguo’ were 10 years old, and the cherries were harvested from the Beijing Academy of Agriculture and Forestry Sciences, located in Beijing. The fruit samples from the different developmental periods were selected according to the anthesis time, being harvested in the small green (SG), mid-green (MG), big green (BG), de-greening (DG), yellow (YW), initial red (IR), full red (FR), and dark red (DR) phases. For the tissue-specific expression analysis, the stems, leaves and flowers were collected from same ‘Zaodaguo’ trees. The mature leaves and stem segments were collected when the leaves were dark green and fully unfolded. The flowers were sampled when they were fully blooming. The samples were transported to the laboratory, then immediately frozen in liquid nitrogen and stored at −80 °C.

The ABA, MeJA, and GA treatments were applied to fruit samples 19–21 days after anthesis. Fruit samples of the same size as the ‘Zaodaguo’ samples were taken back to the laboratory for hydroponic treatment and incubated in the dark for 1 day to eliminate field heat. Then, these fruits treated with exogenous hormones. The concentration of ABA was 100 μmol/L, the MeJA concentration was 200 μmol/L, and the GA concentration was 200 μmol/L. Water was used for the control treatment. The hormone stock solution was prepared using anhydrous ethanol, and the same volume of anhydrous ethanol was added to the control treatment to eliminate errors. After 5 days of the above treatment, the samples were then immediately frozen in liquid nitrogen and stored at −80°C. All treatments were performed with three biological replicates.

### 4.2. Identification of SPL Genes in Sweet Cherry

The protein sequence of 16 AtSPLs were downloaded from the Arabidopsis genome database (https://www.arabidopsis.org/)(accessed on: 12 February 2022). PavSPL protein sequences were searched in the sweet cherry genome database (http://cherry.kazusa.or.jp/) (accessed on: 2 December 2022). The molecular weights, isoelectric points (pIs), and grand average of hydropathicity (GRAVY) values of the PavSPL proteins were calculated using the ExPASy website (https://web.expasy.org/protparam/) (accessed on: 21 June 2022).

### 4.3. Sequence Alignments, Phylogenetic Analyses, and Gene Structure Analysis

We used ExPASy (https://web.expasy.org/protparam/) (accessed on: 21 June 2022) to compute the physical and chemical parameters of each protein sequence. Multiple sequence alignment was carried out using DNAMAN V9.0. https://www.lynnon.com/dnaman.html (accessed on: 18 December 2022). The phylogenetic trees were constructed using MEGA 7.0 software with the neighbor-joining (NJ) method and the bootstrap test was replicated 1000 times. Domains were identified with the SMART (http://smart.embl-heidelberg.de) (accessed on: 19 June 2022) and Pfam (http://pfam.xfam.org) (accessed on: 13 November 2022) programs. The sequence logos of the conserved domains were generated using WebLogo (http://weblogo.berkeley.edu/logo.cgi) (accessed on: 19 November 2022). Furthermore, MEME (http://meme-suite.org/) (accessed on: 13 October 2022) was used to search for motifs in all *SPL* genes. A structure analysis of the *PavSPL* genes was performed using the Gene Structure Display Server (GSDS) (http://gsds.gao-lab.org/) (accessed on: 1 October 2022).

### 4.4. Cis-Acting Element Prediction and Chromosomal Localization Analysis

Cis-acting elements within the *PavSPL* gene promoters were analyzed using the PlantCARE online program (http://bioinformatics.psb.ugent.be/webtools/plantcare/html/) (accessed on: 30 October 2022). TBtools v1.106. https://github.com/CJ-Chen/TBtools/releases (accessed on 2 January 2023) [48] was used to map the genes to chromosomes and visualize these results.

### 4.5. Expression Analysis of PavSPLs

The total RNA was extracted using the RNA extraction kit (Huayueyang Biotechnology, Beijing, China). One microgram of the total RNA was used for the cDNA synthesis using a reverse transcription kit (TaKaRa Biotechnology, Dalian, China). The primers for *SPL* genes were designed using DNAMAN software and tested for specificity using BLAST on the genomics website. All primers of *PavSPL* genes used for the quantitative RT-PCR (qRT-PCR) assay are listed in Supplementary Table S2. The qRT-PCR assay was performed in the presence of SYBR Premix Ex Taq (Kangwei Century Biotechnology, Beijing, China). The reactions were incubated in a Rotor Gene Q Machine (Qiagen, Germany). *PavActin* was screened as internal reference gene for sweet cherry [46]. The relative expression value was quantified using the 2^−∆∆*C*T^ method [49].

### 4.6. Subcellular Localization Analysis

For the colocalization of PavSPL4 and PavSPL7, the full-length PavSPL4 and PavSPL7 sequences without the termination codon were amplified from cDNA from ‘Zaodaguo’ fruit. Each amplification product was a fusion expression with a green fluorescence protein (GFP) label inserted into the pCAMBIA1302 vector under the control of theCaMV35S promoter. The vectors were transformed into the *Agrobacterium tumefaciens* strain GV3101. The primers are listed in Supplementary Table S3.

*N. benthamiana* plants were grown in a plant growth chamber at 24 °C under 16 h/8 h light/dark conditions. These plants can be used as experimental materials at about two months of age. The *Agrobacterium* microbial concentrations were transiently transformed in *N. benthamiana* leaves. The agroinfiltrated leaves were photographed 48 h after transformation. Then, observations with an Olympus laser-scanning fluorescence instrument were performed as described in [50].

## 5. Conclusions

In our study, we identified 12 *PavSPL* genes in the sweet cherry genome database. We systematically studied the gene structures and expression patterns of *PavSPL* genes at different developmental periods and with ABA, GA, and MeJA hormone treatments in sweet cherry fruit. The expression levels of several *PavSPL* genes increased or decreased in response to various hormone treatments. Our results demonstrated that the *PavSPL* genes play important roles in fruit development and ripening. In conclusion, our genome-wide study analysis of the *SPL* gene family lays the foundation for the study of the ripening mechanism in non-respiratory climacteric fruit.

## Figures and Tables

**Table 1:** The information on the *SPL* gene family in *Prunus avium* L.

Gene Name	Accession Number	Protein /AA	Chrom.	Chr Start	Chr End	MW (Da)
*PavSPL1*	*Pav_sc0000065.1_g080.1*	1034	Chr1	6612478	6619865	115029.50
*PavSPL2*	*Pav_sc0000065.1_g070.1*	1009	Chr1	6620405	6626786	112292.00
*PavSPL3*	*Pav_sc0000091.1_g140.1*	316	Chr1	18093845	18097381	34624.93
*PavSPL4*	*Pav_sc0000091.1_g350.1*	443	Chr1	18230101	18234634	48283.35
*PavSPL5*	*Pav_sc0000120.1_g190.1*	434	Chr2	12784392	12787547	47451.18
*PavSPL6*	*Pav_sc0001080.1_g880.1*	217	Chr3	21876290	21878055	25097.53
*PavSPL7*	*Pav_sc0001305.1_g910.1*	162	Chr4	3003020	3004866	18563.10
*PavSPL8*	*Pav_sc0000600.1_g500.1*	1069	Chr4	5991293	5997912	118373.64
*PavSPL9*	*Pav_sc0000975.1_g210.1*	162	Chr4	13389781	13391094	18440.52
*PavSPL10*	*Pav_sc0000740.1_g280.1*	401	Chr5	11637365	11640898	43899.78
*PavSPL11*	*Pav_sc0000358.1_g620.1*	559	Chr5	15069897	15073721	61412.26
*PavSPL12*	*Pav_sc0001280.1_g530.1*	383	Chr6	17835832	17840241	41097.72

AA means amino acid residues. Chrom. means chromosomal location. MW means molecular weight.

**Table 2:** Calculation of Ka and Ks ratios of duplicated *PavSPL* gene pairs.

Gene 1	Gene 2	Ka	Ks	Ka/Ks	Gene Duplication
*PavSPL3*	*PavSPL6*	0.462	1.551	0.298	Segmental
*PavSPL3*	*PavSPL10*	0.406	2.133	0.190	Segmental
*PavSPL6*	*PavSPL10*	0.400	NaN	NaN	Segmental
*PavSPL7*	*PavSPL9*	0.343	1.379	0.249	Segmental

Ka means non-synonymous substitution rate. Ks means synonymous substitution rate. Ka/Ks > 1 is considered a positive selection effect. Ka/Ks = 1 is considered a neutral selection. Ka/Ks < 1 is considered a purifying selection effect. Segmental means that the gene might arise from segmental duplication. NaN means not a number.

## Data Availability

Not applicable.

## Supplementary Materials

The following supporting information can be downloaded at: https://www.mdpi.com/article/10.3390/ijms24032880/s1.

## References

[1] Riechmann J.L., Ratcliffe O.J. (2000). A genomic perspective on plant transcription factors. Curr Opin Plant. Biol..

[2] Klein J., Saedler H., Huijser P. (1996). A new family of DNA binding proteins includes putative transcriptional regulators of the Antirrhinum majus floral meristem identity gene SQUAMOSA. Mol. Gen. Genet..

[3] Cardon G., Hohmann S., Klein J., Nettesheim K., Saedler H., Huijser P. (1999). Molecular characterisation of the *Arabidopsis* SBP-box genes. Gene.

[4] Salinas M., Xing S., Hohmann S., Berndtgen R., Huijser P. (2012). Genomic organization, phylogenetic comparison and differential expression of the SBP-box family of transcription factors in tomato. Planta.

[5] Xiong J., Zheng D., Zhu H., Chen J., Na R., Cheng Z. (2018). Genome-wide identification and expression analysis of the SPL gene family in woodland strawberry Fragaria vesca. Genome.

[6] Li J., Hou H., Li X., Xiang J., Yin X., Gao H., Zheng Y., Bassett C.L., Wang X. (2013). Genome-wide identification and analysis of the SBP-box family genes in apple (*Malus* × *domestica* Borkh.). Plant. Physiol. Biochem..

[7] Song S., Zhou H., Sheng S., Cao M., Li Y., Pang X. (2017). Genome-wide organization and expression profiling of the SBP-Box gene family in Chinese jujube (*Ziziphus jujuba* Mill.). Int J. Mol. Sci..

[8] Yang Z., Wang X., Gu S., Hu Z., Xu H., Xu C. (2008). Comparative study of SBP-box gene family in *Arabidopsis* and rice. Gene.

[9] Yamasaki K., Kigawa T., Inoue M., Tateno M., Yamasaki T., Yabuki T., Aoki M., Seki E., Matsuda T., Nunokawa E. (2004). A novel zinc-binding motif revealed by solution structures of DNA-binding domains of *Arabidopsis* SBP-family transcription factors. J. Mol. Biol..

[10] Birkenbihl R.P., Jach G., Saedler H., Huijser P. (2005). Functional dissection of the plant-specific SBP-domain: Overlap of the DNA-binding and nuclear localization domains. J. Mol. Biol..

[11] Gandikota M., Birkenbihl R.P., Hohmann S., Cardon G.H., Saedler H., Huijser P. (2007). The miRNA156/157 recognition element in the 3’ UTR of the *Arabidopsis* SBP box gene SPL3 prevents early flowering by translational inhibition in seedlings. Plant. J..

[12] Schwarz S., Grande A.V., Bujdoso N., Saedler H., Huijser P. (2008). The microRNA regulated SBP-box genes SPL9 and SPL15 control shoot maturation in *Arabidopsis*. Plant. Mol. Biol..

[13] Wang L., Yu P., Lyu J., Hu Y., Han C., Bai M.Y., Fan M. (2021). BZR1 physically interacts with SPL9 to regulate the vegetative phase change and cell elongation in *Arabidopsis*. Int. J. Mol. Sci..

[14] Zhang Y., Schwarz S., Saedler H., Huijser P. (2007). SPL8, a local regulator in a subset of gibberellin-mediated developmental processes in *Arabidopsis*. Plant. Mol. Biol..

[15] Gou J., Felippes F.F., Liu C., Detlef W., Wang J. (2011). Negative regulation of anthocyanin biosynthesis in *Arabidopsis* by a miR156-targeted SPL transcription factor. Plant. Cell.

[16] Chuck G., Whipple C., Jackson D., Hake S. (2010). The maize SBP-box transcription factor encoded by tasselsheath4 regulates bract development and the establishment of meristem boundaries. Development.

[17] Manning K., Tor M., Poole M., Hong Y., Thompson A.J., King G.J., Giovannoni J.J., Seymour G.B. (2006). A naturally occurring epigenetic mutation in a gene encoding an SBP-box transcription factor inhibits tomato fruit ripening. Nat. Genet..

[18] Eriksson E.M., Bovy A., Manning K., Harrison L., Andrews J., De Silva J., Tucker G.A., Seymour G.B. (2004). Effect of the colorless non-ripening mutation on cell wall biochemistry and gene expression during tomato fruit development and ripening. Plant. Physiol..

[19] Lai T., Wang X., Ye B., Jin M., Chen W., Wang Y., Zhou Y., Blanks A.M., Gu M., Zhang P. (2020). Molecular and functional characterization of the SBP-box transcription factor SPL-CNR in tomato fruit ripening and cell death. J. Exp. Bot..

[20] Liu H., Shu Q., Lin-Wang K., Allan A.C., Espley R.V., Su J., Pei M., Wu J. (2021). The PyPIF5-PymiR156a-PySPL9-PyMYB114/MYB10 module regulates light-induced anthocyanin biosynthesis in red pear. Mol. Hortic..

[21] Li X., Hou Y., Xie X., Li H., Li X., Zhu Y., Zhai L., Zhang C., Bian S. (2020). A blueberry MIR156a-SPL12 module coordinates the accumulation of chlorophylls and anthocyanins during fruit ripening. J. Exp. Bot..

[22] Han Y., Gao H., Chen H., Fu C. (2019). The involvement of papaya CpSBP1 in modulating fruit softening and carotenoid accumulation by repressing CpPME1/2 and CpPDS4. Sci. Hortic..

[23] Zhao X., Qu D., Wang L., Gao Y., An N., Wang A., Li Y., Yang J., Wu F., Su H. (2022). Genome-wide identification of cysteine-rich receptor-like kinases in sweet cherry reveals that PaCRK1 enhances sweet cherry resistance to salt stress. Plant. Cell Rep..

[24] Li Q., Chen P., Dai S., Sun Y., Yuan B., Kai W., Pei Y., He S., Liang B., Zhang Y. (2015). PacCYP707A2 negatively regulates cherry fruit ripening while PacCYP707A1 mediates drought tolerance. J. Exp. Bot..

[25] Gong Y., Fan X., Mattheis J.P. (2002). Responses of ‘Bing’ and ‘Rainier’ sweet cherries to ethylene and 1-methylcyclopropene. J. Amer. Soc. Hort. Sci..

[26] Tijero V., Teribia N., Munoz P., Munne-Bosch S. (2016). Implication of abscisic acid on ripening and quality in sweet cherries: Differential effects during pre- and post-harvest. Front. Plant. Sci..

[27] Kumar R., Khurana A., Sharma A.K. (2014). Role of plant hormones and their interplay in development and ripening of fleshy fruits. J. Exp. Bot..

[28] Chockchaisawasdee S., Golding J.B., Vuong Q.V., Papoutsis K., Stathopoulos C.E. (2016). Sweet cherry: Composition, postharvest preservation, processing and trends for its future use. Trends Food Sci. Tech..

[29] Correia S., Schouten R., Silva A.P., Goncalves B. (2017). Factors affecting quality and health promoting compounds during growth and postharvest life of sweet cherry (*Prunus avium* L.). Front. Plant. Sci.

[30] Zhang D., Han Z., Li J., Qin H., Zhou L., Wang Y., Zhu X., Ma Y., Fang W. (2020). Genome-wide analysis of the SBP-box gene family transcription factors and their responses to abiotic stresses in tea (*Camellia sinensis*). Genomics.

[31] Hou H., Yan X., Sha T., Yan Q., Wang X. (2017). The SBP-box gene VpSBP11 from Chinese wild Vitis is involved in floral transition and affects leaf development. Int J. Mol. Sci..

[32] Liu M., Sun W., Ma Z., Huang L., Wu Q., Tang Z., Bu T., Li C., Chen H. (2019). Genome-wide identification of the SPL gene family in tartary buckwheat (Fagopyrum tataricum) and expression analysis during fruit development stages. BMC Plant. Biol..

[33] Tan H., Song X., Duan W., Wang Y., Hou X. (2015). Genome-wide analysis of the SBP-box gene family in Chinese cabbage (*Brassica rapa* subsp. pekinensis). Genome.

[34] Cai C., Guo W., Zhang B. (2018). Genome-wide identification and characterization of SPL transcription factor family and their evolution and expression profiling analysis in cotton. Sci Rep..

[35] Shen X., Zhao K., Liu L., Zhang K., Yuan H., Liao X., Wang Q., Guo X., Li F., Li T. (2014). A role for PacMYBA in ABA-regulated anthocyanin biosynthesis in red-colored sweet cherry cv. Hong Deng (*Prunus avium* L.). Plant. Cell Physiol..

[36] Qi X., Liu C., Song L., Li M. (2020). PaMADS7, a MADS-box transcription factor, regulates sweet cherry fruit ripening and softening. Plant. Sci..

[37] Jin W., Wang H., Li M., Wang J., Yang Y., Zhang X., Yan G., Zhang H., Liu J., Zhang K. (2016). The R2R3 MYB transcription factor PavMYB10.1 involves in anthocyanin biosynthesis and determines fruit skin colour in sweet cherry (*Prunus avium* L.). Plant. Biotechnol. J..

[38] Chen W., Yu Z., Kong J., Wang H., Li Y., Zhao M., Wang X., Zheng Q., Shi N., Zhang P. (2018). Comparative WGBS identifies genes that influence non-ripe phenotype in tomato epimutant Colourless non-ripening. Sci. China Life Sci..

[39] Cui M., Wang C., Zhang W., Pervaiz T., Haider M.S., Tang W., Fang J. (2018). Characterization of Vv-miR156: Vv-SPL pairs involved in the modulation of grape berry development and ripening. Mol. Genet. Genom..

[40] Leng P., Yuan B., Guo Y. (2014). The role of abscisic acid in fruit ripening and responses to abiotic stress. J. Exp. Bot..

[41] Guo J., Wang S., Yu X., Dong R., Li Y., Mei X., Shen Y. (2018). Polyamines regulate strawberry fruit ripening by abscisic acid, auxin, and ethylene. Plant. Physiol..

[42] Santner A., Calderon-Villalobos L.I., Estelle M. (2009). Plant hormones are versatile chemical regulators of plant growth. Nat. Chem. Biol..

[43] Kuhn N., Ponce C., Arellano M., Time A., Sagredo B., Donoso J.M., Meisel L.A. (2020). Gibberellic acid modifies the transcript abundance of ABA pathway orthologs and modulates sweet cherry (*Prunus avium*) fruit ripening in early- and mid-season varieties. Plants (Basel).

[44] Dong H., Yan S., Jing Y., Yang R., Zhang Y., Zhou Y., Zhu Y., Sun J. (2021). MIR156-targeted SPL9 is phosphorylated by SnRK2s and interacts with ABI5 to enhance ABA responses in *Arabidopsis*. Front. Plant. Sci..

[45] Zhang H., Zhao X., Li J., Cai H., Deng X.W., Li L. (2014). MicroRNA408 is critical for the HY5-SPL7 gene network that mediates the coordinated response to light and copper. Plant. Cell.

[46] Wang Y., Zhai Z., Sun Y., Feng C., Peng X., Zhang X., Xiao Y., Zhou X., Wang W., Jiao J. (2021). Genome-wide identification of the B-BOX genes that respond to multiple ripening related signals in sweet cherry fruit. Int. J. Mol. Sci..

[47] Jia H.F., Chai Y.M., Li C.L., Lu D., Luo J.J., Qin L., Shen Y.Y. (2011). Abscisic acid plays an important role in the regulation of strawberry fruit ripening. Plant. Physiol..

[48] Chen C., Chen H., Zhang Y., Thomas H.R., Frank M.H., He Y., Xia R. (2020). TBtools: An integrative toolkit developed for interactive analyses of big biological data. Mol. Plant..

[49] Livak K.J., Schmittgen T.D. (2001). Analysis of relative gene expression data using real-time quantitative PCR and the 2(-Delta Delta C(T)) Method. Methods.

[50] Sparkes I.A., Runions J., Kearns A., Hawes C. (2006). Rapid, transient expression of fluorescent fusion proteins in tobacco plants and generation of stably transformed plants. Nat. Protoc..

