# Supplementary material for: Genome-Wide Identification of the SQUAMOSA Promoter-Binding Protein-like (SPL) Transcription Factor Family in Sweet Cherry Fruit
Source: Int J Mol Sci. 2023 Feb 2;24(3):2880. doi: 10.3390/ijms24032880 (PMC9917470; doi:10.3390/ijms24032880)
Supplement: Supplementary file 1 [file ijms-24-02880-s001.zip › ijms-2175288-supplementary.pdf]

Supplementary Materials:

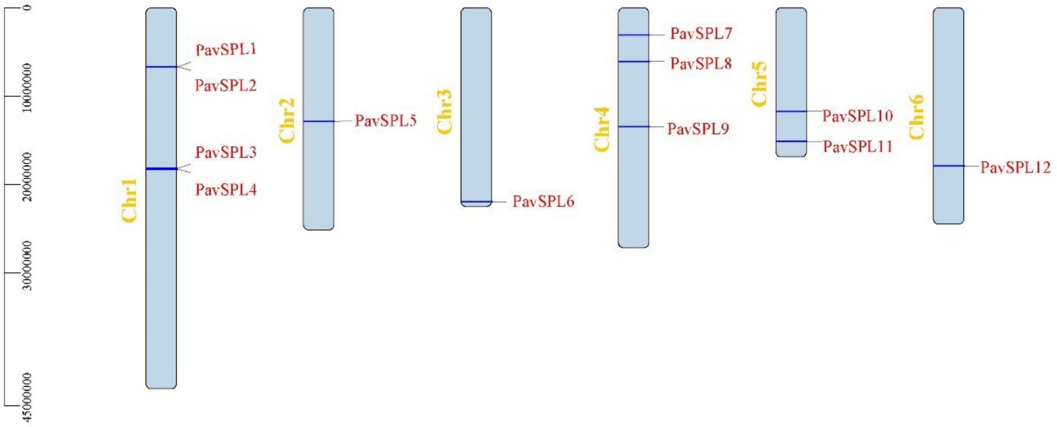

Figure S1: Chromosome distribution of *PavSPL* genes. The scale on the left is in megabases (Mb).

Table S1 Summary genes. information of sweet cherry (*PavSPL*), *Arabidopsis* (*AtSPL*), tomato (*SlySBP* and *CNR*), apple (*MdSBP*), rice (*OsSPL*) and strawberry (*FvSPL*).

Name	**Accession Number**	
AtSPL1	*At2g47070*	[1]
AtSPL2	*At5g43270*	
AtSPL3	*At2g33810*	
AtSPL4	*At1g53160*	
AtSPL5	*At3g15270*	
AtSPL6	*At1g69170*	
AtSPL7	*At5g18830*	
AtSPL8	*At1g02065*	
AtSPL9	*At2g42200*	
AtSPL10	*At1g27370*	
AtSPL11	*At1g27360*	
AtSPL12	*At3g60030*	
AtSPL13	*At5g50570*	
AtSPL14	*At1g20980*	
AtSPL15	*At3g57920*	
AtSPL16	*At1g76580*	
OsSPL1	*Os01g18850*	[1]
OsSPL2	*Os01g69830*	
OsSPL3	*Os02g04680*	
OsSPL4	*Os02g07780*	
OsSPL5	*Os02g08070*	
OsSPL6	*Os03g61760*	
OsSPL7	*Os04g46580*	
OsSPL8	*Os04g56170*	
OsSPL9	*Os05g33810*	
OsSPL10	*Os06g44860*	
OsSPL11	*Os06g45310*	
OsSPL12	*Os06g49010*	
OsSPL13	*Os07g32170*	
OsSPL14	*Os08g39890*	
OsSPL15	*Os08g40260*	
OsSPL16	*Os08g41940*	
OsSPL17	*Os09g31438*	
OsSPL18	*Os09g32944*	
OsSPL19	*Os11g30380*	
FvSPL1	*mrna00196.1*	[2]
FvSPL2	*mrna29726.1*	

FvSPL3	*mrna06692.1*	
FvSPL4	*mrna22888.1*	
FvSPL5	*mrna22889.1*	
FvSPL6	*mrna04565.1*	
FvSPL7	*mrna06295.1*	
FvSPL8	*mrna26117.1*	
FvSPL9	*mrna29149.1*	
FvSPL10	*mrna22660.1*	
FvSPL11	*mrna17738.1*	
FvSPL12	*mrna01338.1*	
FvSPL13	*mrna04784.1*	
FvSPL14	*mrna04786.1*	
CNR	*Solyc02g077920*	[3]
SlySBP12a	*Solyc01g068100*	
SlySBP12b	*Solyc05g053240*	
SlySBP2	*Solyc04g045560*	
SlySBP6c	*Solyc12g038520*	
SlySBP10	*Solyc05g015510*	
SlySBP13	*Solyc05g015840*	
SlySBP6a	*Solyc03g114850*	
SlySBP8a	*Solyc10g018780*	
SlySBP8b	*Solyc01g090730*	
SlySBP3	*Solyc10g009080*	
SlySBP6b	*Solyc05g012040*	
SlySBP15	*Solyc10g078700*	
SlySBP4	*Solyc07g053810*	
SlySBP7	*Solyc01g080670*	
MdSBP1A	*MDP0000861601*	[4]
MdSBP1B	*MDP0000156994*	
MdSBP2	*MDP0000149339*	
MdSBP3	*MDP0000263766*	
MdSBP4A	*MDP0000146640*	
MdSBP4B	*MDP0000778465*	
MdSBP5	*MDP0000803116*	
MdSBP6	*MDP0000262141*	
MdSBP7	*MDP0000170630*	
MdSBP8	*MDP0000865739*	
MdSBP9	*MDP0000158607*	
MdSBP10	*MDP0000171877*	
MdSBP11	*MDP0000246046*	
MdSBP12	*MDP0000155354*	
MdSBP13	*MDP0000919693*	

MdSBP14	*MDP0000919694*
MdSBP15	*MDP0000180408*
MdSBP16	*MDP0000180409*
MdSBP17	*MDP0000271587*
MdSBP18	*MDP0000297978*
MdSBP19	*MDP0000210138*
MdSBP20	*MDP0000589558*
MdSBP21	*MDP0000193702*
MdSBP22	*MDP0000119458*
MdSBP23	*MDP0000249364*
MdSBP24	*MDP0000176265*
MdSBP25	*MDP0000195461*
MdSBP26	*MDP0000142582*
MdSBP27A	*MDP0000255162*
MdSBP27B	*MDP0000538106*

Table S2 The primers sequences for qRT-PCR.

Gene Name	Forward primer sequence (5′-3′)	Reverse primer sequence (5′-3′)
PavSPL1	GGAAATGGGACGGTGATC	CCCTTCTTCGTTTCTCCAAT
PavSPL2	ACGGAGAAGGGCTGTTTT	CAGCCTTACAGTCCTCCACC
PavSPL3	TTCAATCTGGCCAGCTCA	GAAACATGCCCTGGAAATG
PavSPL4	CTGGGGTATTGAAGGGGA	CCCAACTGAAGAACTGACGG
PavSPL5	GCCACTGACAACAACTCATTCT	CGCCGTAGAAACTAGAGTGC
PavSPL6	GGAGCAGCAAAAACAGGAA	TGTGTACAGCAGGAGGAGAATC
PavSPL7	GATCTGGTGCAGGAGGGT	GGAACCTGCTACATTGCTGGC
PavSPL8	CTGCCCCAATTTTCATTCA	CTCACGTTGTCCCAGTCC
PavSPL9	GTCTCCACCATGTTGCCA	CCTCTTTGCTTCGTCGAAC
PavSPL10	GGCACAGTTGATGGGTCTAG	CCACTGTAAGCTGCTCTTGAC
PavSPL11	GCTTTGATGGGTTGGGAG	CACCATAAACCCTGCCACT
PavSPL12	CTTTGAGGATGCGGTTCTC	GAGCCAAATTCACACCCC
PavACTIN	CCAGGGCTGTGTTTCCTTCTA	ATGATCTGCGTCATCTTTTCT

Table S3 The primers sequences for plasmid construction.

Gene Name	Forward primer sequence (5′-3′)	Reverse primer sequence (5′-3′)
PavSPL4-1302	GACTCTTGACCATT-AGATCTGATGAATTCTGTGTTGCTGATG	GTGAAAAGTTCTTCTCCTTTACTAGTATTGAATTGGTT-GGTCTGATAA
PavSPL7-1302	GACTCTTGACCATT-AGATCTGATGGAATCAAACAGAGCTCAT	GTGAAAAGTTCTTCTCCTTTACTAGTTTCTCCATGA-TAATCAGAGGAG

1. Yang, Z.; Wang, X.; Gu, S.; Hu, Z.; Xu, H.; Xu, C. Comparative study of SBP-box gene family in Arabidopsis and rice. *Gene* **2008**, *407*, 1-11, doi:10.1016/j.gene.2007.02.034.
2. Xiong, J.; Zheng, D.; Zhu, H.; Chen, J.; Na, R.; Cheng, Z. Genome-wide identification and expression analysis of the SPL gene family in woodland strawberry Fragaria vesca. *Genome* **2018**, *61*, 675-683, doi:10.1139/gen-2018-0014.
3. Salinas, M.; Xing, S.; Hohmann, S.; Berndtgen, R.; Huijser, P. Genomic organization, phylogenetic comparison and differential expression of the SBP-box family of transcription factors in tomato. *Planta* **2012**, *235*, 1171-1184, doi:10.1007/s00425-011-1565-y.
4. Li, J.; Hou, H.; Li, X.; Xiang, J.; Yin, X.; Gao, H.; Zheng, Y.; Bassett, C.L.; Wang, X. Genome-wide identification and analysis of the SBP-box family genes in apple (Malus x domestica Borkh.). *Plant Physiol Biochem* **2013**, *70*, 100-114, doi:10.1016/j.plaphy.2013.05.021.
